# Retraction of “UBE2C, Directly Targeted by miR-548e-5p, Increases the Cellular Growth and Invasive Abilities of Cancer Cells Interacting with the EMT Marker Protein Zinc Finger E-box Binding Homeobox 1/2 in NSCLC”

**DOI:** 10.7150/thno.50254

**Published:** 2020-07-25

**Authors:** Dan Jin, Jiwei Guo, Yan Wu, Jing Du, Xiaohong Wang, Jiajia An, Baoguang Hu, Lingqun Kong, Weihua Di, Wansheng Wang

**Affiliations:** 1 Clinical Medical Laboratory, Binzhou Medical University Hospital, Binzhou, 256603, P.R. China; 2 Cancer research institute, Binzhou Medical University Hospital, Binzhou, 256603, P.R. China; 3 Department of Thyroid and Breast Surgery, Binzhou Medical University Hospital, Binzhou, 256603, P.R. China; 4 Department of Clinical Laboratory, Binzhou Medical University Hospital, Binzhou, 256603, P.R. China; 5 Department of Gastrointestinal Surgery, Binzhou Medical University Hospital, Binzhou, 256603, P.R. China; 6 Department of Hepatobiliary Surgery, Binzhou Medical University Hospital, Binzhou, 256603, P.R. China

The Editor-In-Chief of Theranostics, in consultation and agreement with the editorial board members, retracts the article “UBE2C, Directly Targeted by miR-548e-5p, Increases the Cellular Growth and Invasive Abilities of Cancer Cells Interacting with the EMT Marker Protein Zinc Finger E-box Binding Homeobox 1/2 in NSCLC” 1 on the basis of questions related to irregularities within several figures. The questions about data irregularities also raise questions about the conclusions within the paper.

## References

[1] Jin D, Guo J, Wu Y, Du J, Wang X, An J (2019). UBE2C, Directly Targeted by miR-548e-5p, Increases the Cellular Growth and Invasive Abilities of Cancer Cells Interacting with the EMT Marker Protein Zinc Finger E-box Binding Homeobox 1/2 in NSCLC. Theranostics.

